# BATMAN: A Brain-like Approach for Tracking Maritime Activity and Nuance

**DOI:** 10.3390/s23052424

**Published:** 2023-02-22

**Authors:** Alexander Jones, Stephan Koehler, Michael Jerge, Mitchell Graves, Bayley King, Richard Dalrymple, Cody Freese, James Von Albade

**Affiliations:** Washington Business Office, Riverside Research Institute, Arlington, VA 22202, USA

**Keywords:** dark ships, ship behavior, data fusion, artificial intelligence, neural networks, satellite imagery, AIS data, geospatial intelligence

## Abstract

As commercial geospatial intelligence data becomes more widely available, algorithms using artificial intelligence need to be created to analyze it. Maritime traffic is annually increasing in volume, and with it the number of anomalous events that might be of interest to law enforcement agencies, governments, and militaries. This work proposes a data fusion pipeline that uses a mixture of artificial intelligence and traditional algorithms to identify ships at sea and classify their behavior. A fusion process of visual spectrum satellite imagery and automatic identification system (AIS) data was used to identify ships. Further, this fused data was further integrated with additional information about the ship’s environment to help classify each ship’s behavior to a meaningful degree. This type of contextual information included things such as exclusive economic zone boundaries, locations of pipelines and undersea cables, and the local weather. Behaviors such as illegal fishing, trans-shipment, and spoofing are identified by the framework using freely or cheaply accessible data from places such as Google Earth, the United States Coast Guard, etc. The pipeline is the first of its kind to go beyond the typical ship identification process to help aid analysts in identifying tangible behaviors and reducing the human workload.

## 1. Introduction

With the constant increase in global trade and commerce, maritime activity across the globe is on the rise. Shipping lanes are becoming more crowded, fishing vessels are traveling farther as fisheries deplete, and yachts are increasingly becoming common property among the wealthy [1,2,3]. These increases in traffic create opportunities for illicit or dangerous activity to occur, such as illegal fishing, trafficking, piracy, infrastructure tampering, etc. [4,5,6]. Organizations such as government enforcement agencies are very interested in preventing these activities from happening. Luckily, industry has responded to these desires in the form of commercially available, open-source geospatial intelligence. This increasing availability of information allows for heightened monitoring of ever-increasing global maritime activity. Imagery in the visible and infrared bands are available from vendors such as Planet or Maxar Technologies, or aggregate sources such as Google Earth. Synthetic aperture radar (SAR) imagery is available from companies such as ICEYE or Capella Space. Automatic Identification System (AIS) data, which acts as easily accessible telemetry and descriptive data that ships broadcast worldwide, is available from entities such as Spire or ORBCOMM. All of these forms of data allow for detection of illicit activity by tracking where ships are and what they are doing in correspondence to other vessels around them.

The data modalities provided by these vendors do not simply show illicit activity at sea. The data is raw and must be analyzed by algorithms or artificial intelligence (AI) to determine what exactly is occurring within it. Some companies do provide some form of analytics with their data when purchased [7,8], but it is often surface-level and is only meant to provide further context for analysts, algorithms, or AI to further discern what might be occurring within the data.

Bad actors at sea are obviously aware of these various forms of surveillance and observation being conducted by government agencies and commercial entities. Many bad actors will seek to avoid detection by visual or AIS means by concealing themselves. For example, in visual spectrum image data, the bad actor might choose to conduct their activity at night or during inclement weather. For AIS data, the bad actor might choose to spoof their reported location or not broadcast their AIS data at all. Many boats that do not meet the legal minimum requirements for AIS transmitters do not possess them at all. These types of actors are colloquially known as dark ships and are seeking simply not to be detected. That, however, does not mean all dark ships are bad. In some cases, their reasons for concealment could be accidental or completely benign. A cargo ship might be concealing its AIS transmissions when traversing a seaway known for high rates of piracy. A fishing vessel could be conducting fishing at night, since nighttime is when things such as squid are most easily caught [9]. A lot of things happen at sea, and it should be up to the analysts and their AI algorithms to determine if they are bad or good behaviors. 

Since AIS was internationally adopted as a standard safety measure in 2002 [10], many publications have investigated using AIS information to detect dark ships [11,12]. The definition of dark ship that these publications use can vary from paper to paper, but the term typically describes ships that are failing to transmit AIS messages at a rate that falls within international maritime law compliance or are blatantly spoofing messages [13,14,15]. What these studies lack, however, is any form of cross-referenced data that can corroborate their claimed observations based on the AIS data. AIS data only tells part of the story, so claims purely based on this single data modality can be considered weak or incomplete.

More recently, some other publications have begun looking into verifying ship positions and linking AIS data to specific ships found in satellite imagery [16,17,18,19,20]. This process is known as ship-pairing, and often involves some sort of algorithmic approach to pairing AIS messages received at discrete points in time with satellite imagery taken at separate points in time. These studies perform the task of ship-pairing, but often do not dare to go much further beyond discerning any form of higher level of behavior from the information extracted. Studies such as [16] do discuss some manually derived speculation about what certain dark ships found within the ship-pairing process might be doing, but none of it is obtained using algorithms or AI. Without any AI assistance when analyzing the high volumes of data in geospatial intelligence, an analyst’s job is still slow, tedious, and cumbersome. Having an AI that goes one step further than the ship-pairing process could help identify tangible behaviors or activity occurring within multi-modal information.

Image and AIS data are both fantastic starting points for fusing multiple forms of data together to form a more complete picture of what is occurring within maritime space. However, simple images and entries in an AIS data table do not tell the full picture of what might be happening in a specific area. External contextual info such as weather, tidal information, and air pollution can give dynamic information regarding the local situation about factors that might be influencing ship behavior. Likewise, more general geographic information such as location of nearby pipelines and undersea cables, exclusive economic zones and protected ecosystem boundaries, proximity to ports, and the AIS legal requirements of each nation can provide further context as to activities ships might be engaged in. Further fusing these forms of contextual info in with the image and AIS data modalities would help further bolster the behavior claims an AI could make for the ships it observes. 

**Figure 1:**
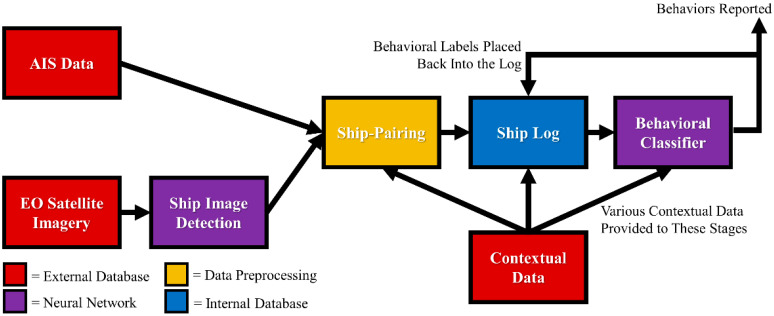
Data flow of the BATMAN framework.

The work conducted in this paper proposes a framework that uses a combination of both traditional algorithms and AI to fuse multiple data modalities together, identify ships within that data, and classify their behavior. This framework, known as a “brain-like approach for tracking maritime activity and nuance,” or BATMAN, is one that uses a pair of neural networks to identify ships in imagery and then classify their behavior. In-between the networks, a sequence of preprocessing algorithms operates to identify ships before having the ships’ behavior classified by the second neural network (Figure 1). The entire system is deployed to an Amazon Web Services (AWS) framework which can adequately handle the size of data expected in a geospatial intelligence problem space. 

The work here will first describe the types of datasets that were utilized to train the neural networks used and to verify operation of the preprocessing algorithms. It will then discuss how each of the neural networks and algorithms operated at every stage of the processing pipeline. How the system was trained and deployed to AWS is then described, followed by the results obtained at each stage of the pipeline. A discussion section then explores what the results at each stage mean within BATMAN, and how these results impact the future design direction for the project. Finally, a conclusion summarizing results is included at the end of the work. 

## 2. Materials and Methods

### 2.1. Datasets

#### 2.1.1. Imagery

With the increase of available high-resolution remote sensing ship datasets [21,22,23,24], these data can be used in the pipeline to physically identify ships at specific locations [24,25]. Due to the zoom restrictions of remote orbital imagery, ship detection is often considered a problem of small object detection (SOD) [26,27]. 

The ShipRSImageNet [21] dataset combines multiple collections of high-resolution optical images to be used for ship detection. The dataset features over 3000 images containing over 17,000 ships in total, in both open water and costal locations. Horizontal and vertical bounding boxes are offered, along with four levels of labeling. The simplest labeling has two classes (ship or dock), while the most complex labeling scheme has 50 total labels, covering various types of commercial ships and warships. This work only considered images from the set with the same ground sample distance (gsd) of 1.07, which were then rescaled to a common size of 1256 by 728.

One other dataset used for training the ship detector is the Google Earth EO Dataset, which was created for BATMAN specifically. A total of 600 satellite images were captured using Google Earth [28], all within the US and Cuban EEZs from 2018–2021. These dates and regions were chosen so that the images would correspond to Marine Cadastre [29] AIS data, which will be discussed in Section 2.1.2. At least one ship is present in 300 of the 600 images of this dataset. The remaining 300 images contain no ships.

**Figure 2:**
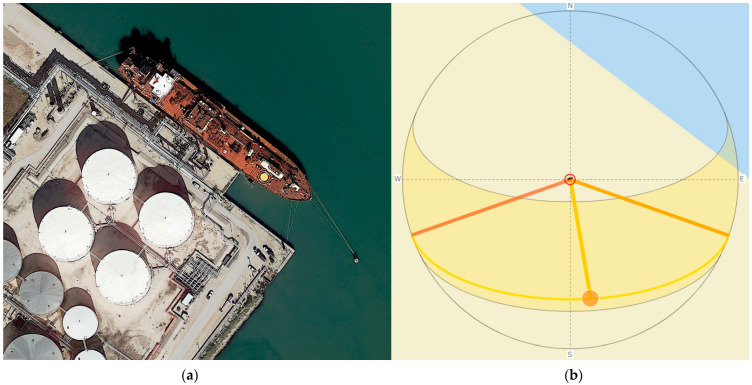
A demonstration of the “sundial” calculation in the Google Earth EO dataset, demonstrating the time of day at which the image was captured: (**a**) a sample image from the Google Earth EO dataset, with visible shadows cast by the ship and circular structures; (**b**) the position of the sun as viewed using the SunCalc.org tool on the corresponding date and location of the image, which is provided by Google Earth. The position of the sun is chosen to correspond to the position of the shadows in the image. For this example, the image was captured near Corpus Christi, TX on 31 January 2020. Using the “sundial” technique, the image was determined to be captured at approximately 12:15 p.m. local time.

The latitude and longitude, meters per pixel, and date information for each image were collected from Google Earth each time an image was captured. However, the time of day for each image was not reported by Google Earth and needed to be estimated. A tool that demonstrates the location of the sun based on date, time of day, and location, called SunCalc [30] allowed for time-of-day prediction to within an hour. Shadows cast by ships, trees, buildings, channel markers, and other structures were used as “sundials” and compared with the location of the sun shown by the SunCalc tool. This “sundial” calculation is demonstrated in Figure 2.

#### 2.1.2. AIS Data

Data was obtained via compressed zip files for each day of US coastal AIS transmissions from Marine Cadastre [31], which were locally stored for each day. The Marine Cadastre AIS data was chosen for this work since it is freely available and contains a high volume of AIS messages. The popular python library “pandas” was used for treating tables consisting of millions of rows of transmissions, where each row contains information such as time stamp, ship MMSI, latitude, longitude, ship type, etc. The daily tables of transmissions were directly read into pandas’ data frames, which were cleaned, and the data types were optimized, sorted in ascending order for the two columns MMSI and time stamp and finally stored as .feather files. Although this file format has limitations not present for other formats, such as “pickle” or HDF5, its speed and small size justified this choice. Moreover, the complexity of the data frames did not preclude using .feather format. 

The AIS transmission time stamps are completely asynchronous; some ships broadcast at a high rate (every few seconds) and others broadcast very infrequently (intervals between transmissions can be several days or more). Data on all ships’ positions at the time of the image is necessary to merge EO or other synchronous data such as SAR or drone footage with the AIS data stream. Simple linear interpolation for all numeric features was used, such as latitude, longitude, speed over ground, etc.; however, for other non-continuous data such as vessel status or vessel type, the value closest in time to that of the image was used.

To successfully time-interpolate AIS transmissions, one transmission must exist before the current transmission, and one must exist after the current transmission. This interpolation can be complicated when the ship’s transmission rate is low, or if the requested time interpolation is either before (or after) the first (last) transmission of that day since the data is grouped into 24 h periods. For example, interpolating the positions at the start or end of the day is not possible unless data from previous or subsequent days is available. This complication is resolved by utilizing continuous time sequences (e.g., weeks or months), where for each day and MMSI collected the first and last transmission is placed into a data frame. When performing time interpolation for any given day, the daily data frame with the appended data frame of first and last transmissions is used. If the latter spans enough days, the interpolation requirement can be met, except for very few edge cases. These edge cases include cases in which the ship (or its AIS transmitter) is new, or the ship is no longer transmitting. 

To significantly increase computation speed, all grouping operations (grouping by operation in pandas) are eschewed in favor of creating a monotonically increasing index on the sorted data frame that wraps the MMSI and time stamp together. This joint index is created by multiplying the MMSI number by the maximum time stamp value and adding the actual time stamp value. To meet the value limitations imposed by 64-bit integers, key values of a MMSI column categorization scheme are utilized.

#### 2.1.3. Static Contextual Data

To facilitate the classification of maritime vessel behavior, locations and coordinates were trial-tested with several different API services before coming to a consensus on the selected API services. Contextual data was broken down into two categories: a static contextual database with data that does not change at all, and the dynamic contextual database which provides more historical context, along with the ability to forecast.

These static contextual files helped to build a larger narrative of each ship’s behavior. Each one can provide insight to a larger picture. EEZ’s helped to define maritime boundaries and laws within those boundaries. The expectation is that a ship should and will adhere to the laws of whichever country’s EEZ it is residing in. One can use these various protected zones to gather whether a ship is engaging in illicit behavior to hide its location to circumscribe the jurisdiction of international and local laws. A ship can spoof its transmission to appear somewhere other than where it is. Regarding fishing vessels, this is not always an illegal act, but rather the business practice of protecting a known “sweet spot,” known only to that captain, crew or company [32].

The static contextual database consisted of GeoJSON files. (GeoJSON is an open standard format designed for representing simple geographical features, along with their non-spatial attributes.) The files that contained underwater sea cables and their anchor points to the mainland came from TeleGeography [33]; those designating Marine Protected Areas came from the National Oceanic Atmospheric Administration [34] (NOAA), the United Nations Educational, Scientific and Cultural Organization (UNESCO) and texts mapping other areas came from United Nations Environment Program World Conservation Monitoring Centre (UNEP-WCMC) and the International Union for Conservation of Nature (IUCN), both through Protected Planet [35]. The static contextual database also contained GeoJSONs of Exclusive Economic Zones (EEZ) and their intersections and overlapping claims, as described on maps from MarineRegions.org [36]. Global Energy Monitor also provided us with GeoJSON files of undersea oil and gas pipelines [37].

In addition to the collected GeoJSON files, the United Nations charters and conventions related to maritime law were investigated for their ratification status by country, along with a governing set of rules applied to all countries [38]. The database features information based on all the countries that currently fly flags of convenience (FoC) on the ocean and their ratification status as to all these legal and maritime conventions. While most of the provisions have been adopted by states of the UN, several countries exclude one or more of the conventions. This database also contains the Paris and Tokyo Memorandum of Understanding on Port State Control White/Gray/Blacklist [39,40]. This list classifies countries on a color status based on the number of infractions cited against ships flying FoC for that state. A comprehensive list of all database sources can be found in Table 1.

#### 2.1.4. Dynamic Contextual Data

The dynamic contextual database is generated from World Weather Online and OpenWeatherMap [41,42]. The World Weather Online database is gathered from worldwide historical weather over the course of three years. This API provided not only historical weather in the middle of the ocean, but also astronomical data with regards to lunar activity, and tidal information in reference to swells and significant wave height, as well as daily high and low changes in the tides. OpenWeatherMap provides worldwide air pollution data. This API returned the concentration of particulate matter of 2.5 and 10 microns, and gas concentrations of CO, NO, NO_2_, O_3_, SO_2_, and NH_3_. The contextual data could allow one to detect how weather impacts the behavior of ships, and using OpenWeatherMap API, one can then extrapolate the possibility of a ship’s circumventing the rules pertaining to sulfurbased fuels in accordance with the MARPOL 73/78 regulation amended in IMO 2020 [42]. Pollution data could also be used as a tool for helping the classifier detect general areas of ship activity or movement.

### 2.2. BATMAN Pipeline

#### 2.2.1. Objectives and Structure

The overall objective for the BATMAN framework is to intake multiple modalities of geospatial intelligence data and identify ships within them. Then, those identified ships will be integrated with additional contextual data to classify their behavior as previously described in Figure 1. Prior to the image and AIS data being fused in the ship-pairing process, the AIS data undergoes some preprocessing techniques to extract more pertinent information from its contents. Additionally, the image data is passed through an object detection neural network to physically identify ships within the imagery. This fused data is then passed to the Ship Log where the identified ships and their associated time stamps are stored. These ships are then routed to the behavioral classifier to determine what they are precisely doing at each point in time. That information is then rerouted back into the Ship Log so it can be used in later classifications.

#### 2.2.2. Data Format 

Contextual data was retrieved from various sources, and therefore came in multiple formats. Legal information was provided in CSV files. Weather and tidal data were acquired as JSON responses from API calls. Regional information was provided in GeoJSON format. A list of each dataset along with their data formats can be found in Table 2.

#### 2.2.3. The Ship Log

The Ship Log is the datastore for all available ship information to be ingested by the behavioral classifiers. AIS information is stored alongside YOLO ship detections that have been processed by the ship pairing algorithm. All instances of ship detections that were successfully paired with AIS messages will contain the corresponding AIS information in their entries within the Ship Log. Ship detections that are not paired with any AIS-identified ships are left with empty values for any information that would be reported in AIS messages, such as MMSI, speed over ground, cargo type, and draft. Each entry in the Ship Log also contains information indicating the ship’s legal requirements to send AIS messages.

#### 2.2.4. Pipeline Data Flow

The BATMAN pipeline streams data from different sources depending on the type of data being collected. AIS data, for example, is streamed from the Marine Cadastre API. The imported data is loaded into the DynamoDB database Ship Log for further processing. Furthermore, EO images are streamed from the Google Earth EO database and passed through the YOLO detection algorithm. If ships are detected within the EO image stream, the resultant output is paired with data from the Ship Log. If a pair does not result from the ship pairing algorithm, the output then passes through a legality filter lambda function to determine whether a ship is legally required to transmit AIS data given its geographic location. If a ship is not legally bound to transmit AIS data, the output from the YOLO neural network is added to the Ship Log. Once these processes have completed, another algorithm takes as input the entries from the Ship Log and adds contextual data from the dynamic context database. The ship info is then fed into the behavioral classifier neural network, which then determines ship behaviors based on characteristics of the Ship Log entries. 

### 2.3. Algorithms

#### 2.3.1. YOLO and Other Methods for Ship Detection

Ship detection was the most widely investigated specific area of research of Deep Learning for Object Detection in Earth Observational (EO) data, according to a recent survey of the field [44]. Hoeser et al. [44,45] continued into the second part of this survey to discuss some of the nuances of ship detection, compared to other object detection, and note its common use in application for novel advancements in Deep Learning literature. They also note the difficulty of in-shore (or costal) imagery compared to off-shore (open ocean) image and suggest the use of land-sea masks from elevation models to help determine coastlines, and eliminate false positives of ship-like objects on land [46]. Segmentation [47,48,49,50,51,52,53], and the use of rotated bounding boxes [54] have also been found to be improvements directed towards ship detection.

Modern object detection models can often be split into two architectures, two-stage detectors, and one-stage detectors. Although two-stage detectors are also popular in application, this review will focus on one-stage detection, such as the You Only Look Once (YOLO) [55,56,57], Single Shot Detection (SSD) [58], and RetinaNet [59] algorithms. These one-shot detectors offer high accuracy, while being suited for more edge environments that require low power for inference (e.g., space orbital deployment). YOLO is a widely used network, created and popularized by Redmon dating back to their initial network [55]. With this increase in popularity of ship detection as the application for object detection algorithms, many proposed additions to one-stage detectors are showcased around ship detection datasets, especially YOLO [60,61,62,63,64,65,66,67,68]. 

Newer models exist, such as the EfficientDet-D7 [69], which proves to be a promising new model using single-shot detection [45]; improvements over ship detection results from older version of EfficientDet-D0 [70] can be expected with these newer architectures. A recent survey of ship detection algorithms noted the general lack of work analyzing the spatiotemporal data after detection, and instead focused on the performance of the ship detection [44].

#### 2.3.2. Ship-Pairing Algorithm

A key goal for BATMAN is matching AIS data to ships found in images, and determining which ships are not properly broadcasting AIS, (i.e., running dark). For a given image, the ships’ locations are determined in terms of pixels and latitude/longitude, as described previously. The positions of all ships are interpolated for the image’s acquisition time and optimized via distance-based matching. Conceivably, matching could also involve additional features such as the ship’s heading, size or type recorded in the AIS transmission and these can be determined from image analysis; however, AIS transmissions of ship size or type are unreliable due to operator error. In many cases the sizes are not entered at all or entered improperly (width and length are frequently interchanged, or wrong units other than meters are used). A general mean interval measurement, *I_m_*, is used to minimize the difference in meters between the image’s ship positions and those from the time-interpolated AIS data. 

The ship-pairing function has two parameters limiting the temporal and spatial range: the threshold for the distance between messages and imaged ships, *T_m_*, and the threshold for the time between messages and imaged ships, *T_t_*. Additionally, one internal hyperparameter defines clustering: *ε* sets the distance between points of the same cluster. Clustering reduces the search space of possible matches and significantly improves computation times. An additional parameter is the maximum number of permutations, *P_max_*, which limits the number of ship-pairing assignments considered. Setting *P_max_* to 1 is greedy in the sense of purely locally optima. 

The principle behind clustering ships in an image is for reasonable clustering such that AIS candidates for different clusters will be non-overlapping. For example, consider two ship clusters that are separated by a large distance (e.g., 100 km). The pairing for each cluster can be treated independently, because the pools of possible AIS ships are distinct, and thus the number of permutations considered is significantly reduced.

The optimization involves considering pairing permutations between the ships of a cluster and the AIS candidate ships within a *T_m_* distance and having a transmission within *T_t_* of the image’s time. The first permutation to be considered is greedy, and subsequent permutations are variations where the pairing distance increases from the local optimum.

To help determine an ideal setting for these hyperparameter values, a performance metric, *r*, is defined, which is a ratio between the number of ships paired and a normalized mean interval.

(1) r=spImTm

In Equation (1), *s_p_* represents the number of ships being paired, *I_m_* is the mean interval between identified ships and their associated AIS messages, and *T_m_* is the interval threshold (in meters). For situations in which runtime is also a performance factor, a second metric, *r_t_*, was also defined that also further divides *r* by the runtime, *t*.

(2) rt= rt

#### 2.3.3. AIS Data Feature Engineering

Since the amount of AIS data being processed is quite large, a guiding principle of the AIS preprocessing is to reduce file space. This process involves eliminating duplicate dates, judicious use of data typing, and minimizing computational load. For example, the downloaded data from Marine Cadastre, which is a compressed zip file, does not get uncompressed as a csv file, and instead is directly read into a pandas data frame using *pandas.read()*_csv. After some cleaning (removing duplicate rows or removing rows missing key features such as MMSI, time stamp, latitude, longitude, etc.), the data type is optimized to decrease data size. This reduction involves using categorical data types when appropriate, such as strings (e.g., ship names), converting floats to integers by converting NaNs to −1 (when possible), and converting string date times to pandas’ date–time objects. Depending on the precision required, the values are reduced from float64 to float32 or float16, or int64 is reduced to int32, int16, or even int8. Care is taken for numerical calculations to increase the data to either float64 or int64 for preventing over-flows.

One challenge using vectorized geometries for geotagging based on latitude/longitude from AIS transmissions is the computational load, which for millions of rows, can lead to long computing times. To eliminate these computations, the vectorized data is rasterized at a sufficient resolution (about 0.001 degrees, which is 1 km or less) using makegeocube from the geocube API and saved as an array with to_raster. Using the rasterization as a look-up table reduced the computational burden by orders of magnitude as compared to determining regions of vectorized geo-data. Moreover, the geometry data was removed from the geo-file (either shapely or geopandas) and saved as a much smaller file using the binary file .feather format developed by Apache Arrow. One limitation of this approach is that it implicitly assumes that all geometries are non-overlapping, a consideration which needs to be addressed in the future.

#### 2.3.4. Behavioral Classifier Data Ingestion

As data is uploaded to S3, AWS Lambda functions automatically retrieve and save both dynamic and static contextual data for future ingestion by the behavioral classifier. For dynamic context, World Weather Online is queried for weather and tidal data for a given latitude and longitude at the appropriate time. Pollution levels for a number of atmospheric particulates are retrieved using the OpenWeatherMap.org API. This information is saved to a new data frame that is loaded during training. For static context, each data point is compared to lists of exclusive economic zones, submarine cables, UNESCO marine World Heritage sites, and fisheries to determine important context about each ship’s location. This information is again stored in a new data frame. 

#### 2.3.5. Behavioral Classifier Neural Network Approaches

Two neural network types, dense (~2,000,000 parameters) and convolutional (~32,000,000 parameters) [71] were used. Both models were relatively small with the dense model having three dense layers and an output layer, and the convolutional model having a dense layer, three convolutional layers, a final dense layer, and an output layer. Both models used the same input data and augmentations, output shape, and loss function to attempt to identify various ship behaviors. As these behaviors are not mutually exclusive, each model needs the ability to output multiple predictions. To accomplish this, a custom loss function (shown below) was used (where *i* is an index of the output array and *n* is the total number of predicted behaviors).

(3) $$\overset{n}{\underset{i=0}{\sum }}-(y\_true_{i}*\log\left(y\_pred_{i}\right)+\left(1-y\_true_{i}\right)*\log\left(1-y\_pred_{i}\right))$$

This custom loss function takes the standard binary cross-entropy loss and applies it to each of the output behaviors independently to allow for multi-label classification. 

During training many of the samples required a significant amount of cleaning to allow the network to train effectively. This adjustment was needed due to several factors, including fake transmissions, corrupt data, and irregular transmissions causing calculated metrics, such as distance and speed between transmissions, to have large variances. This mainly involved removing duplicate AIS transmissions and transmissions missing MMSI or latitude and longitude. To accommodate these value ranges, a large portion of the data was clipped. To allow the model to still accommodate these extreme values, without causing the model to diverge in training due to these extreme values, the data was normalized to a range that was physically realistic (e.g., −1 -> 0 m/s and +1 -> −50 m/s), then the upper bound of that normalized column was clipped at +2. Additionally, several columns contained categorical data that was encoded using a base-2 encoding scheme.

For this phase of the research, these networks showed sufficient results, and further research will look to incorporate more advanced architectures such as Tabnet [72] and Tabformer [73].

#### 2.3.6. Behavioral Classifier Traditional Approaches

Three traditional machine learning algorithms were developed for the study to compare the neural network approaches against established behavioral classifiers. The algorithms used were ensemble method (Extremely Randomized Trees, or ERT), a classification and regression tree (CART) method, and a gradient boosted method (Gradient Boosted Trees). The choice to include algorithms from three different categories was due, in part, to the decision to baseline several different techniques against the neural network classifier, as well as to incorporate results from various categories of traditional approaches. Each of the classifiers utilized the TensorFlow library to incorporate the data processing pipeline, preprocessing the data and storing the results as TensorFlow-records. The Extremely Randomized Trees algorithm includes a maximum number of 100 trees and a sparse oblique split axis. The GBT model includes a maximum number of 149 trees, as that was the optimized number of trees needed to perform with the greatest accuracy. The random seed for both ERT and GBT models utilized an initial random seed of 1000, as compared to the default random seed of 1234. All models included the initial input and output shape of the neural networks to comparatively incorporate dimensionalities. 

#### 2.3.7. Generating Behavioral Truth Labels for Training

Behavioral truth labels were generated by labeling the data with ten different behaviors, based entirely on Marine Cadastre AIS data. The entire month of January 2022 was time-resampled with an hourly frequency. The behaviors were: wandering, transshipment, coastal loitering, offshore loitering, illegal fishing, concealed illegal fishing, competitive fishing, generic loitering, docked, and tampering (Table 3). Ship Log entries could have none of these behaviors or one or many of these behaviors.

An underlying feature for many of the behaviors of interest is loitering, which is briefly described here. For loitering, the key parameter is the distance threshold that distinguishes small-scale movement, such as an anchored ship drifting with changing currents or small fluctuations in the GPS transmissions due to the limited precision and lack of directed movement that would be typical of a traveling ship. For the hourly-resampled data, the feature “successive loitering” was defined for a threshold distance of 5 km, as follows: within the hour the ship’s excursions have to be less than 5 km, and for the subsequent hour the distance between both mean positions has to also be less than 5 km. Thus, any ship loitering for several successive hours has to be moving extremely slowly or essentially be stationary. Moreover, vessels engaged in fishing are considered to also be loitering (i.e., they are moving very slowly).

Truth labels for each entry in the Ship Log were generated using contextual info and some of the data features are mentioned in Section 2.3.3. The truth labels were generated by checking for specific values present within the data. The classification of “Concealed Illegal Fishing” deserves further explanation, as, in principle, knowing the location of a concealed (dark) vessel is impossible using AIS alone. However, the trajectories are interpreted at hourly intervals, which can in fact result in trajectories intersecting protected fishing grounds. Although the approach for creating labeled data is basic and involves assumptions regarding interpolated waypoints, it provides a foundation for proving BATMAN as a concept. The technique paves the way as a baseline comparison for more complex data generation techniques in the future, such as synthetic data generation or unsupervised learning schemes. Multi-modal geospatial intelligence datasets do not exist in easily accessible places, particularly ones that contain labeled data. Creating one in this manner is a critical first step in demonstrating BATMAN’s capabilities.

### 2.4. Deployment Platforms

#### 2.4.1. NVIDIA DGX-1 Training

For model training and tuning, a Nvidia DGX-1 that featured 8 Tesla V100 GPUs, an Intel Xeon CPU model E5-2968 v4 at 2.20 GHz, with 528 GBs of RAM, was used. The behavioral classification pipeline worked inside of a Docker container: lightweight standalone software packages containing every file needed to run an application (code, runtime, system tools, etc.). Shared data (results, logs, datasets, etc.) was then kept in shared storage, which could then be mounted to a container. 

#### 2.4.2. AWS Deployment

**Figure 3:**
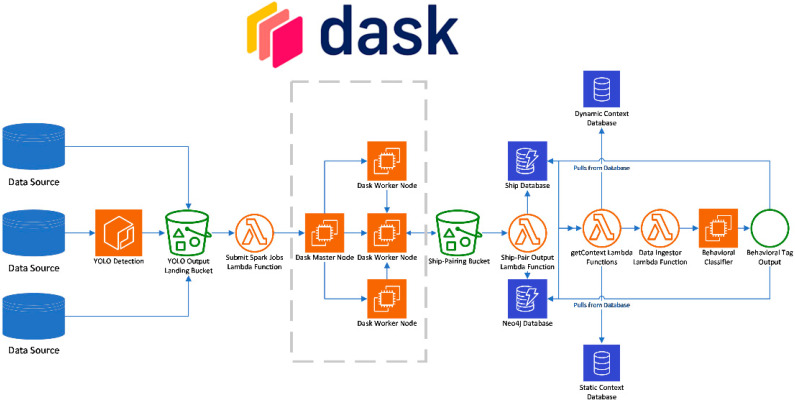
AWS structure of BATMAN.

The computing architecture of the pipeline was deployed using Amazon Web Services cloud computing resources. A diagram showing an AWS version of the pipeline shown in Figure 1 is shown in Figure 3. Retrieval and storage of data follows a traditional extract, transform, and load (ETL) process [74], for future applications in real-time edge cases. ETL pipelines extract data from source, transform the data into a usable structure for algorithms, and then load the data into the necessary storage mechanism. To simulate part of the use case of a neuromorphic chip placed on an edge device, the completed YOLO model was stored in a Docker container, which was subsequently placed within an Ubuntu EC2 T2 instance. As the EO data is extracted from the source, it streams through the YOLO Docker container and, if a ship is detected, the output is uploaded to an S3 data lake storage bucket. The uploading of this data then triggers an AWS Lambda function to instantiate a transient elastic map reduce (EMR) Dask cluster to pair the EO detection with existing AIS data. The ship-pairing output is stored as a parquet file in another S3 bucket, which itself triggers an additional legality filter AWS Lambda function upon successful upload to the bucket. Once the event passes through the Lambda function, it is stored in a master Ship DynamoDB database. Contextual and Static Data Lambda functions are then triggered for use in the neural network behavioral classification model. 

## 3. Results

### 3.1. Ship Detection and Classification

In place of describing a new novel ship detection algorithm, a brief review of literature over the problem in question is presented. The review is focused only on applications using the YOLO architecture, and is not a comprehensive review for all published work using YOLO for ship detection. Many publications were left out of this review that did not report either mean average precision (mAP), recall, and/or precision.

As Table 4 shows, many variations of YOLO have been used for ship detection problems, with high precision and recall. SAR and EO were the dominant dataset types, with most EO coming directly from partially released satellite datasets. Long et al. [63] and Zhu et al. [67] presented smaller versions of YOLO, which still offered comparable results to larger YOLO models. This review has shown that many novel advancements to Deep Learning in recent years have been applied to the problem of ship detection, and the use of lightweight models can offer edge-based deployment and desirable detection results.

### 3.2. Ship-Pairing Algorithm

**Figure 4:**
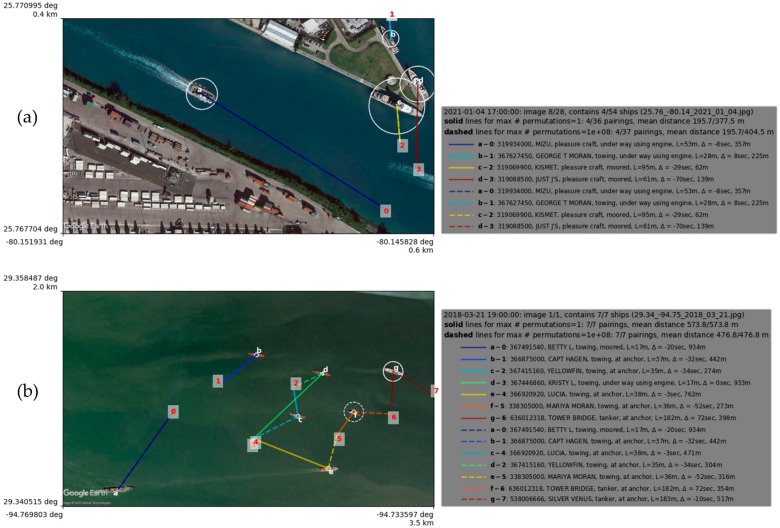
Examples of the ship−pairing algorithm, working against the Google Earth EO dataset and the Marine Cadastre AIS data. Each ship within the image is labeled with a letter, while each AIS message within the confines of the image is labeled by a number. Solid lines represent ship pairings when *P_max_* = 1 (Greedy Approach) and dashed lines represent ship pairings when *P_max_* = 10^8^. (**a**) An example in which both the greedy and extensive approaches agree on the ship pairings within the image. All four ships within the image are paired to the same AIS messages by both techniques. (**b**) A more complex example, where discrepancies appear between the two pairing approaches. The cluster of ships on the right-hand side of the image causes the two approaches to attempt to pair AIS messages with different ships.

Parametric studies of the ship-pairing process were performed for a range of the parameters *T_m_*, *T_t_*, and *P_max_*. The analysis paired the ships found in the Google Earth EO dataset with ships found in the Marine Cadastre AIS dataset. The purpose of this study is determining optimal parameters for this type of data in terms of matching the highest number of ships, *s_p_*, with the lowest overall *I_m_* and having a reasonable computation time considering all 600 rows. Two examples of the ship-pairing algorithm in action can be seen in Figure 4.

Results of the ship-pairing analysis are shown in Table 5. When *T_m_* and *T_t_* are both set to 1e4, *r* and *r_t_* are at their highest value. However, *r* is at its maximum value when using the extensive approach, while *r_t_* is highest during the greedy approach. This result means that an improvement is obtained when using the extensive approach, but when considering time as a factor, the additional runtime is negligible.

### 3.3. Truth Label Generation

The AIS dataframes were augmented with labels to match truth labels with ship-paired EO images. For example, spoofed transmissions were identified as having a greater or lesser degree of intended latitude, longitude, and elevation. Furthermore, resampled AIS data was assembled with a global clock, which in turn, further maintained precise measurements relative to behavior truth labels. Neighborhood analysis for the global clock was then conducted to appropriately label the aforementioned ten behavioral classes.

**Figure 5:**
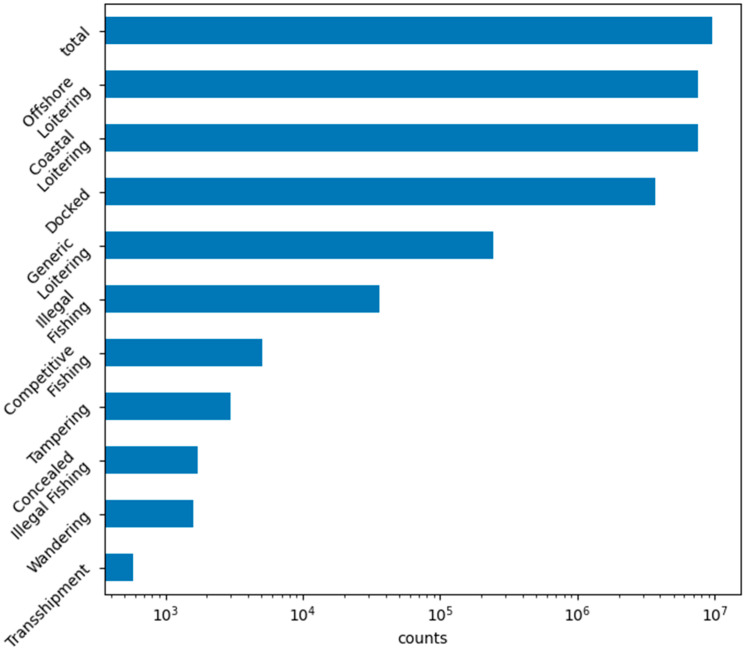
Bar chart showing total counts across the Ship Log that were labeled in the final version to be used for behavior classifier training. Many loitering behaviors were quite common, while other behaviors, such as transshipment and wandering, were quite rare.

The final labels generated are shown in Figure 5. These labels are to be used as truth data for the behavioral classifiers to train against for accuracy evaluation. Many behaviors, such as the various forms of loitering, were quite common. Other behaviors, such as fishing behaviors, existed across a range of occurrence rates. The data used for training primarily encompasses the month of January 2022 and was taken from the Marine Cadastre database.

### 3.4. Behavioral Classifier

**Figure 6:**
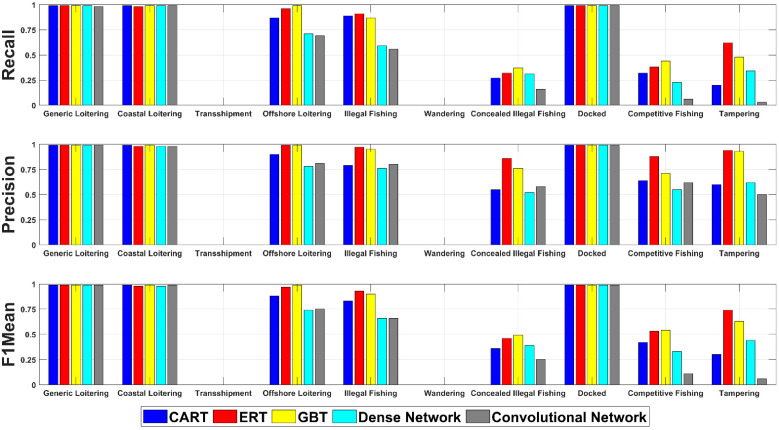
Results for all techniques tested for the behavioral classifier. Recall, precision, and F1Mean scores are shown for all behaviors labeled in the Ship Log. No algorithm was able to detect the rare transshipment and wandering behaviors.

Results for the five algorithms used in the behavioral classifier (three traditional algorithms and two neural networks) are shown in Figure 6. The data shown is the result of having trained the behavioral classifier against a Ship Log containing data specifically from January 2022. Recall, precision, and F1Mean are broken down individually by behavior. 

As can be seen in the figure, the classes with the most samples were predicted significantly more accurately than those with fewer samples. In total there were 441,677 total samples, of which 23,415 had no corresponding behaviors (normal ship behavior) that were split 80:20 for training and testing, respectively. Three behaviors were predicted nearly perfectly by all algorithms, two behaviors were detected with high confidence by the traditional approaches, three behaviors were detected with moderate success by all algorithms, and two behaviors were never predicted. Detailed discussion of these results can be found in Section 4.3. 

## 4. Discussion

### 4.1. Effects of False Negatives and False Positives

During testing, the question of the importance of false negatives (FN) vs. false positives (FP) was asked, in addition to the question of whether the network should be biased towards one or the other. If the case is considered where a FN is transmitting AIS data, the data would still be parsed to the classifier. Notwithstanding this, a heavy bias is now applied to the classifier to label the ship, which can only be detected from AIS transmissions, as possibly spoofing their location. In the case of FP, the classifier would get the inverse of this bias, now towards the ship’s perhaps being dark (i.e., not transmitting information). YOLO models have a harder time classifying smaller ships, and since some regulations allow for smaller vessels not to transmit AIS per SOLAS regulation [26], a FN of a smaller vessel could bypass the incorrect classification of a dark ship.

Since natural conditions such as cloud coverage, weather, and darkness could perhaps shield a ship from being detected by remote electro-optical imagery, FN should be expected more often in cases of non-malicious behavior. Because of this trend, it is instead better to tune classifiers to accept more FN than FP, as other data modalities could still help classify a FN. In the case in which a prediction is very sure (i.e., a high confidence value), but there is no corresponding paired AIS message, then the classifier should be predisposed to report back that there is a ship that is spoofing their AIS. 

When considering the issue of FN, the topic of small ships should certainly be mentioned whenever discussing ship detection. Whether in the image or the AIS domain, small ships are rather difficult to detect. A small ship in satellite imagery might be only a few pixels in size, and many small vessels do not possess AIS transmitters, since they are not legally required to do so. Ensuring that the detection algorithm is tuned to detect small ships is of the utmost importance, since they are the ones least likely to appear within AIS data. In future versions of BATMAN, more advanced image analysis could be utilized to detect small ships within imagery, such as ship wake detection. This type of analysis would allow for detection of ships that are even barely visible within an image for small boats in motion. 

### 4.2. Ship-Pairing Dynamics

**Figure 7:**
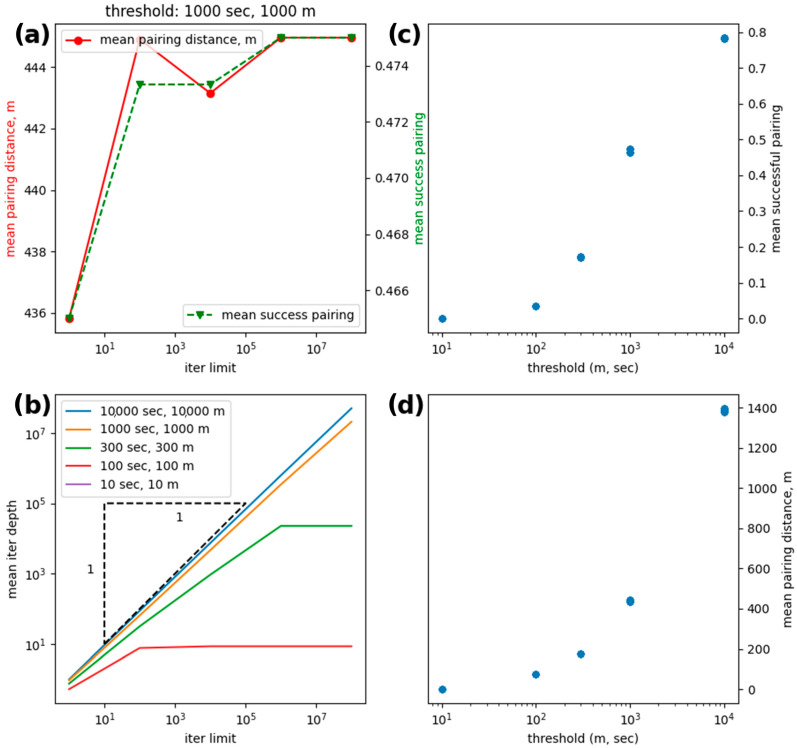
Summary of ship-pairing performance: (**a**) Setting both time and distance thresholds to 1000 s/meters, the dependence of mean pairing distance (left axis) and the mean pairing success (right axis) on the iteration limit: At *P_max_* = 10^4^ the mean pairing distance drops, while the number of pairings stays constant, and at *P_max_* = 10^6^ two more pairings are created, which causes the mean pairing distance to increase. (**b**) Dependence of the mean iteration depth on the iter limit for five sets of thresholds, where the saturation occurs at increasingly greater iter limits with increasing thresholds; (**c**) the pairing success rate increases with thresholds; (**d**) mean pairing distance also increases with threshold.

For all parameter choices, pairing is generally improved by increasing *P_max_*. These improvements are either made in terms of an increased number of pairs or a decreased mean interval distance. Increasing *P_max_* even up to millions of permutations increases computation time by no more than 40 s, or 10%. Moreover, for smaller values of *T_m_* (i.e., <10 km), the number of successful pairs is increased over greedy pairing. For a given time threshold, the number of paired ships is saturated beyond a threshold distance of 10 or more km (469 for 10,000 s, 452 for 1000 s and 441 for 1000 s), which can be achieved by both greedy and more sophisticated approaches. On the other hand, the mean interval distance of these pairings is slightly improved when searching over permutations. Below, the distances required for the number of greedy pairs drops faster than those for permutation pairs. However, when the number of pairs is increased by the permutational search, the mean distance increases by a greater amount than the decrease at high threshold distances. Dynamics of how pairing scales with varying *T_s_* and *T_m_* can be found in Figure 7.

As a final note on ship-pairing, the algorithm currently attempts to optimize the pairing process via various distance calculations and averages across the identified ships in the two data modalities. It does not try and optimize to metrics such as ship size or class. As of now, the Ship Log records the ship class in accordance with the self-reported ship class within the AIS data. However, the ship image detection algorithm could be trained to identify all the ship classes found in AIS data and optimize ship-pairing in accordance with a ship’s class and size in addition to its location.

### 4.3. Behavioral Classification Dynamics

The two most significant training factors varied during training of the neural networks were learning rate scheduling and loss function. As noted in Section 2.3.5, the custom loss function greatly outperformed mean-square error and mean absolute error, especially in classes with fewer labels. Further improvements to this loss function would include class weighting to more heavily weight classes with fewer examples. Additionally, a simple learning rate decay showed significant improvement over a static learning rate.

In general, the traditional approaches performed just as well, if not better, than the neural network approaches tested. In classes where performance was similar, the number of examples in those classes was high enough to the point where all algorithms were able to successfully generalize the behavior. In other cases where behaviors were less common, the neural network approaches failed to perform as well since they did not have as many samples to train against. However, in the cases of transshipment and wandering, the classes were so rare that no algorithm was able to successfully detect the behavior. These scenarios show that techniques such as synthetic data generation or using unsupervised learning schemes in future work could aid in boosting the performance of standard neural networks, such as the dense or convolutional networks. The traditional approaches could also benefit from a higher sample count of the rarer behaviors, which could be made available via synthetic data generation.

When looking at the results from Figure 6 overall, at points, it is a little difficult to tell which algorithm is performing best. The recall, precision, and F1Mean values across all behaviors for each algorithm, were averaged, and the values obtained can be found in Table 6. The top performer across each metric is in bold print.

Overall, ERT performed best on average across all three key metrics. However, as can be seen in Figure 6, there are behaviors where algorithms such as GBT clearly surpass ERT (e.g., offshore loitering). In future versions of BATMAN, an ensemble approach to classifying ship behavior could be considered in which each algorithm is queried with their responses weighted with respect to the reliability of that specific algorithm for the specific behavior. It should also be noted that the algorithm performance shown here could be influenced by how the labeled data was generated for training. If training data is generated differently in future work, the performance of each approach could shift in either a positive or negative direction. Since the data for this work was generated in a rather structured manner via tagging entries in the Ship Log, the structure could be influencing the high performance of the traditional approaches (i.e., CART, ERT, and GBT). If future data is less separable in nature, the neural network approaches could begin to surpass the performance of CART, ERT, and GBT.

Further improvements in the design of the neural networks would look towards architectural improvements and better normalization processes. As noted in Section 2.3.5, Tabformer and Tabnet are two of the leading architectures for classification of tabular data; however, other sequence modeling or transformer architectures could also provide improvements over the current approaches. To improve normalization processes, additional preprocessing steps could be taken, such as creating more intelligent ways for removing obviously corrupt data, creating additional features for values outside given ranges (e.g., speed > 50 m/s), or incorporating other encoding methods to the preprocessing pipeline. Results shown in Figure 6 demonstrate that there is room for improvement across both neural network and traditional approaches, and neural networks such as Tabformer or Tabnet could be helpful in closing the gap on detect rarer behaviors within the Ship Log.

A key conclusion drawn from the behavioral classification process was that most of the behaviors studied were fairly simple to detect to at least some degree. However, the true challenge in a problem space such as classifying ship behavior at a broad scale lies within properly fusing data together that can be digested by a classifier, and then properly labeling that data to reflect real world scenarios. In the field, these algorithms could detect ship behavior using different modalities of data, but if the system remains fixed in the type of data it intakes and how it learns from it, malicious actors at sea could adjust their behavior to further avoid detection. For example, if the ship detection algorithms used against EO imagery have difficulties detecting small ships, and all the criminals using large ships are caught with BATMAN, a survivorship bias could occur where criminals using small vessels are the only ones remaining and are left to flourish. Likewise, if the behavioral classifier is fixed on attempting to classify specific behaviors by specific routes ships run when at sea, but then dynamics of the routes change, those behaviors would either be misidentified or not identified at all. For the long term, it would be critical for a framework such as BATMAN to adopt a form of lifelong learning scheme [80] to its weights where they are constantly considering new data.

Lastly, during behavioral classification, the contextual data most likely plays a minor supplementary role in helping to identify specific ship behavior. Information such as weather data might help the algorithms isolate activity, such as the various forms of loitering or being docked, which could possibly increase in occurrence during times of inclement weather, when seas are rougher. Although this work did not specifically analyze how significant a role contextual data played in behavior classification success, future work aims to do so.

## 5. Conclusions

This work has shown and demonstrated a multi-modal data fusion pipeline that is able to identify ships and classify their behavior. This system, named BATMAN, can fuse satellite image data and AIS data together to identify ships through a ship-pairing process. The ship-pairing process was able to identify 78% of ships present within the images and AIS data (the other 22% could be considered “dark”). These ships can then be classified by ten different behaviors that provide clarity as to their actions. In the cases in which sufficient examples were present within the data for each behavior, the classifier was able to recognize them with high recall and precision. All of this analysis was conducted using an interconnected setup on Amazon Web Services. To the authors’ knowledge, this is the first time a framework to classify maritime activity in such a comprehensive manner has ever been publicly documented. BATMAN and its future iterations could serve as excellent aides for maritime analysts in the public, private, and academic sectors.

Although not comprehensive in its behavioral classification capability, BATMAN serves as a solid foundation with which to begin classifying ship behavior at a more holistic level. In the data modality domain, BATMAN can already cross-validate the existence of ships across the image and AIS domain. Other modalities such as acoustics or radio communications data could greatly enhance BATMAN in the future to allow it to detect ships that might be difficult to detect in the image and AIS domains. In the behavioral classification domain, generating data that is more dynamic and possesses a wider range of behaviors is critical to ensuring that BATMAN can fully understand the maritime environment and all possible scenarios. Both corporations and government organizations that have vested interests in the maritime domain should begin thinking about ship activity at the comprehensive level that BATMAN does. By providing more context to ships’ activities, BATMAN could make the world’s oceans safer places for all seafarers.

## Figures and Tables

**Table 1:** Database Charters and References.

Geographical (Static)	Atmospheric (Dynamic)	Legal (Static)
NOAA [34]	World Weather Online [41]	UN Rules [38]
Protected Planet [35]	OpenWeatherMap [42]	Port State Control Lists [39,40]
MarineRegions [36]		IMO 2020 [43]
Global Energy Monitor [37]		
TeleGeography [33]		

**Table 2:** Data formats.

Dataset	Format
Marine Cadastre AIS	Compressed ZIP files containing CSV files
Google Earth EO	JPEG files and XML files
Static contextual	CSV and GeoJSON files
Dynamic contextual	JSON formatted API response

**Table 3:** Labeled Behaviors within Ship Data and Their Conditions.

Behavior	Definition	Additional Conditions
Wandering	Cargo ships in seldom traveled areas	More than 10 km from coast, >50% waypoints untraveled
Transshipment	Pairs of ships at sea loitering in close proximity	More than 10 km from coast, proximity < 20 m
Coastal Loitering	Loitering near the coast and away from ports	Less than 10 km from coast
Offshore Loitering	Ships loitering out of port away from the coast	Loitering > 10 km from coast
Illegal Fishing	Fishing vessels loitering in protected zones	-
Concealed Illegal Fishing	Dark fishing vessels loitering in protected zones	-
Competitive Fishing	Dark fishing vessels loitering at sea	-
Generic Loitering	Ships loitering out of port	Located 5 km or further from port
Docked	Ships loitering in port	Within 5 km of port
Tampering	Diving or dredging vessels loitering near pipelines/undersea cables	Within 2 km of pipelines/cables

**Table 4:** Review of results from recent publications using YOLO networks for ship detection. All datasets without a direct citation were published as part of the listed authors’ published work.

Publication	Base Network	Imagery	Dataset	Train Size	Test Size	mAP	Recall	Precision
Khan and Yunze, 2018 [75]	YOLOv2	SAR	Sentinel-1	6003	74	-	95.95%	-
Long et al., 2020 [63]	YOLOv3	SAR	SSDD [76]	878	282	89.72%	-	-
Long et al., 2020 [63]	Tiny-YOLOv3	SAR	SSDD [76]	878	282	88.69%	-	-
Zhang et al., 2020 [77]	YOLOv3	EO + IR	DSDR	1146	738	-	84.62%	78.09%
Hu et al., 2021 [61]	YOLOv4	EO	MASATI [78]	1675	711	91.00%	-	-
Jiang et al., 2021 [62]	YOLOv4	SAR	SSDD [76]	812	348	96.32%	95.96%	96.98%
Tang et al., 2021 [64]	YOLOv5	SAR	GaoFen-3	~10,285	~1714	90.97%	92.65%	70.80%
Zhu et al., 2021 [67]	YOLOv5	SAR	SSDD [76]	812	348	64.90%	97.50%	87.80%
Zhu et al., 2021 [67]	YOLOv5	SAR	HRSID [22]	~3923	~1681	72.00%	94.90%	72.40%
Wang et al., 2022 * [65]	YOLOv5	EO	DIOR [79]	-	-	-	68.20%	-
Wang et al., 2022 * [65]	YOLOv5	EO	CDIOR	-	-	-	81.90%	-
Xu et al., 2022 ** [66]	YOLOv4	EO	WFV ***	-	-	92.32%	90.53%	94.93%
Xu et al., 2022 ** [66]	YOLOv4	EO	PMS ***	-	-	93.07%	90.75%	94.93%

* Incorrect definition of False Positive ** No coastal images used in dataset *** Taken from the GaoFen-1 satellite.

**Table 5:** Ship Pairing Comparison for *ε* = 1 km.

Approach	*T_m_* (m)	*T_t_* (s)	*t* (s)	*s_p_*	*I_m_* (m)	*I_m_/T_m_*	*n*	*r_t_*
Extensive (*P_max_* = 10^6^)	10^4^	10^4^	439.81	469	1342.55	0.13	**3493.35**	7.94
	10^4^	10^3^	460.25	452	1368.31	0.14	3303.35	7.18
	10^4^	10^2^	458.96	441	1485.64	0.15	2968.42	6.47
	10^3^	10^4^	414.34	302	366.59	0.37	823.81	1.99
	10^3^	10^3^	426.71	289	373.24	0.37	774.30	1.81
	10^3^	10^2^	433.04	273	390.81	0.39	698.55	1.61
	10^2^	10^4^	410.65	70	47.31	0.47	147.96	0.36
	10^2^	10^3^	423.82	63	49.02	0.49	128.52	0.30
	10^2^	10^2^	424.94	55	51.60	0.52	106.59	0.25
Greedy (*P_max_* = 1)	10^4^	10^4^	405.53	469	1352.15	0.14	3468.55	**8.55**
	10^4^	10^3^	437.46	452	1376.33	0.14	3284.10	7.51
	10^4^	10^2^	419.27	441	1494.92	0.15	2949.99	7.04
	10^3^	10^4^	405.80	293	342.49	0.34	855.50	2.11
	10^3^	10^3^	412.65	280	348.12	0.35	804.32	1.95
	10^3^	10^2^	425.27	267	375.44	0.38	711.17	1.67
	10^2^	10^4^	442.90	69	46.29	0.46	149.06	0.34
	10^2^	10^3^	410.19	62	47.91	0.48	129.41	0.32
	10^2^	10^2^	405.61	54	50.37	0.50	107.21	0.26

**Table 6:** Average performance values across each algorithm for behavior classification.

Algorithm	Recall	Precision	F1Mean
CART	55.2%	64.5%	57.6%
ERT	**61.5%**	**76.0%**	**65.9%**
GBT	61.2%	73.1%	65.2%
Dense Network	51.5%	61.9%	55.2%
Convolutional Network	44.6%	62.7%	48.0%

## Data Availability

Data and code for all the analysis conducted in this work can be found online via GitHub at https://github.com/RiversideResearchAIML/BATMAN (accessed on 15 February 2023).
